# Supplementary material for: The Advantages of Clinical Nutrition Use in Oncologic Patients in Italy: Real World Insights
Source: Healthcare (Basel). 2020 May 6;8(2):125. doi: 10.3390/healthcare8020125 (PMC7349883; doi:10.3390/healthcare8020125)
Supplement: Supplementary file 1 [file healthcare-08-00125-s001.pdf]

healthcare

MDPI

Article

The Advantages of Clinical Nutrition Use in Oncologic Patients in Italy: Real World Insights

Paolo Pedrazzoli [1,†], Riccardo Caccialanza [2,†,*], Paolo Cotogni [3], Luca Degli Esposti [4], Valentina Perrone [4], Diego Sangiorgi [4], Francesco Di Costanzo [5], Cecilia Gavazzi [6], Armando Santoro [7] and Carmine Pinto [8]

1 Medical Oncology Fondazione IRCCS Policlinico San Matteo and Department of Internal Medicine and Medical Therapy, University of Pavia, 27100, Pavia, Italy; p.pedrazzoli@smatteo.pv.it
2 Clinical Nutrition and Dietetics Unit, Fondazione IRCCS Policlinico San Matteo, 27100, Pavia, Italy
3 Pain Management and Palliative Care, Department of Anesthesia, Intensive Care and Emergency, Molinette Hospital, University of Turin, 10126, Turin, Italy; paolo.cotogni@unito.it
4 Clicon S.r.l., Health Economics and Outcomes Research, 48121, Ravenna, Italy; Luca Degli Esposti: luca.degliesposti@clicon.it (L.D.E.); valentina.perrone@clicon.it (V.P.); diego.sangiorgi@clicon.it (D.S.)
5 Medical Oncology Unit, AOU Careggi, 50134, Firenze, Italy; dicostanzofrancesco@icloud.com
6 Clinical Nutrition Unit Fondazione IRCCS Istituto Tumori Milano,20133, Milan, Italy; cecilia.gavazzi@istitutotumori.mi.it
7 Humanitas Clinical and Research Center IRCCS, Rozzano and Humanitas University, 20089 Milan, Italy; armando.santoro@cancercenter.humanitas.it
8 Medical Oncology Unit, Clinical Cancer Centre IRCCS - AUSL Reggio Emilia, 42122, Reggio Emilia, Italy; carmine.pinto@ausl.re.it
* Correspondence: Correspondence: r.caccialanza@smatteo.pv.it; Tel.: +39-0382-501615
† Both authors contributed equally to this work.

Index

Data Availability Statement 3

Ethics Information 3

Figure S1. Proportions of metastatic patients who had a malnutrition diagnosis among those with or without clinical nutrition. 3

Figure S2. Non-metastatic patients with at least three months of follow-up who had clinical nutrition stratified by time of clinical nutrition administration.............. 4

Table S1. Characteristics of metastatic patients receiving clinical nutrition with at least three months of follow-up stratified by time of clinical nutrition (CN) administration[1]... 5

Table S2. Results from Cox multivariate regression models comparing death risk in metastatic patients receiving early versus late clinical nutrition. 6

Table S3. Differential diagnoses of malnutrition in the cohort of metastatic patients with CN (a) and without CN (b). 7

Table S4. Results from Cox multivariate regression models comparing death risk in malnourished metastatic patients with clinical nutrition versus without clinical nutrition. 8

Table S5. Characteristics of non-metastatic patients who received clinical nutrition stratified by cancer type. 9

Table S6. Results from Cox multivariate regression models on non-metastatic patients comparing death risk in non-metastatic patients receiving early versus late clinical nutrition.. 10

Data Availability Statement

All data used for the current study are available next to CliCon s.r.l. which is the body entitled of data treatment and analysis by Local Health Units.

Ethics Information

The name of the Ethics Committees that approved the study, along with the number/ID of the approvals are:

- Comitato Etico Interprovinciale Area 1 – approved on 5/12/2017 – Parere 142
- Comitato Etico di Bergamo – approved on 17/2/2017 – Studio Nutrizione Parenterale
- Comitato Etico Campania Nord – approved on 13/12/2017 – Prot. N. 301963/ASL
- Comitato Etico di Area Vasta Sud Est – approved on 20/02/2017 – Prot. N. 34
- Comitato Etico Regionale per la sperimentazione clinica della Regione Toscana – approved on 17/07/2017 – Prot N. 191/2017
- Comitato Etico della Asl di Lecce – approved on 6/9/2017 – Verbale N. 10
- Comitato Etico Area Pavia – approved on 31/07/2017 – Prot. N. 20170025740
- Comitato Etico Unico Regionale FVG – approved on 1/8/2017 – Prot. N. 22669
- Comitato Etico per la sperimentazione clinica delle province di Verona e Rovigo – approved on 5/4/2017 – Prot. N. 18523

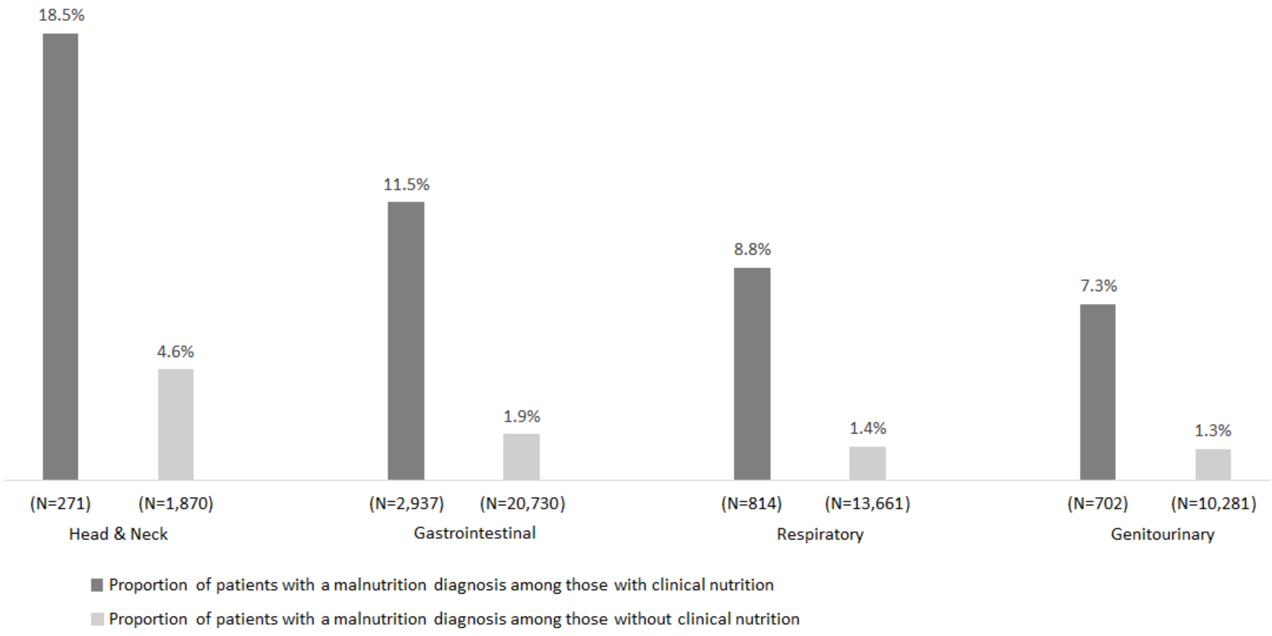

Figure S1. Proportions of metastatic patients who had a malnutrition diagnosis among those with or without clinical nutrition.

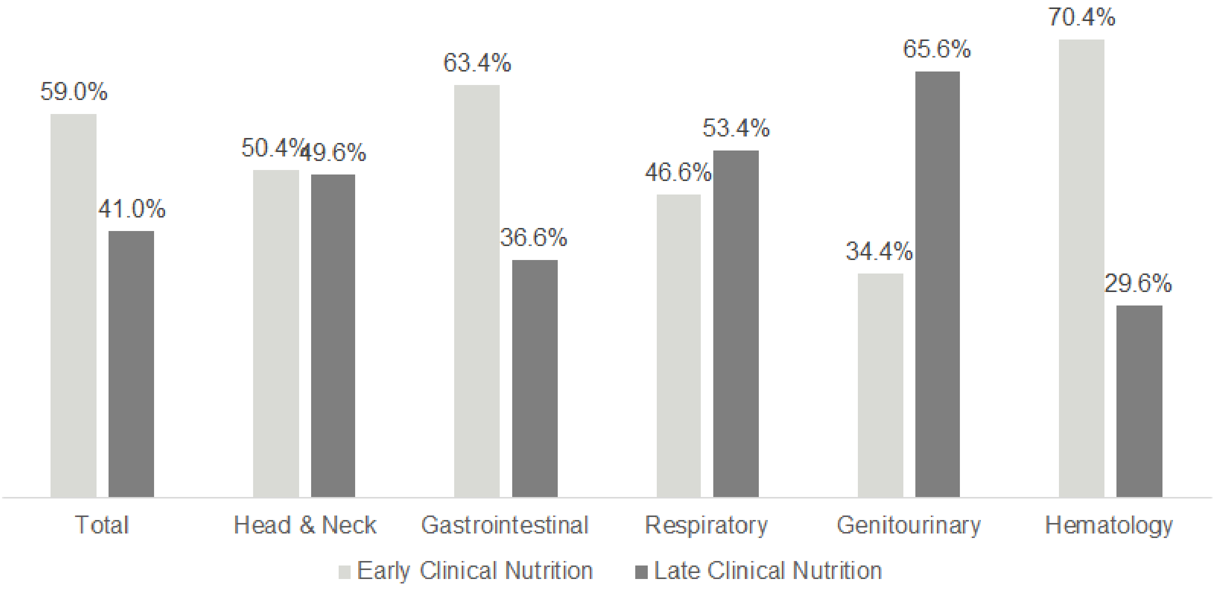

Figure S2. Non-metastatic patients with at least three months of follow-up who had clinical nutrition stratified by time of clinical nutrition administration.

Table S1. Characteristics of metastatic patients receiving clinical nutrition with at least three months of follow-up stratified by time of clinical nutrition (CN) administration[1].

	**Metastatic patients with early CN (*N* = 545)**				**Metastatic patients with late CN (*N* = 1824)**			
	N (%)[2]	**Age Mean (SD)**	**Men** *N* (%)[3]	**mCCI[4] Mean (SD)**	*N* (%)[2]	**Age Mean (SD)**	**Men** *N* (%)[5]	**mCCI[4] Mean (SD)**
All Clusters (*N* = 2369)	545 (23.0%)	68.6 (12.7)	334 (61.3%)	1.0 (0.9)	1,824 (77.0%)	65.3 (12.3)	1,107 (60.7%)	1.0 (1.0)
Head and Neck (*N* = 152)	27 (17.8%)	60.8 (10.4)	20 (74.1%)	0.7 (0.7)	125 (82.2%)	60.8 (11.1)	96 (76.8%)	0.8 (0.8)
Gastrointestinal (*N* = 1478)	408 (27.6%)	69.1 (12.6)	243 (59.6)	0.9 (0.9)	1,070 (72.4%)	65.5 (12.4)	629 (58.8%)	1.0 (1.0)
Respiratory (*N* = 389)	48 (12.3%)	69.0 (10.8)	35 (72.9%)	1.4 (1.1)	341 (87.7%)	65.8 (10.9)	258 (75.7)	1.2 (1.0)
Genitourinary (*N* = 312)	52 (16.7%)	68.6 (13.5)	27 (51.9%)	0.9 (0.9)	260 (83.3%)	66.3 (13.3)	111 (42.7)	1.0 (1.0)
Hematology (*N* = 38)	10 (26.3%)	66.0 (18.9)	9 (90.0%)	1.3 (1.1)	28 (73.7%)	62.0 (18.0)	13 (46.4%)	1.1 (1.2)

[1]Quartiles of time (months) since metastasis diagnosis to first clinical nutrition prescription was calculated on all metastatic patients receiving clinical nutrition; patients were then classified as having an early or late administration when presenting a time since metastasis diagnosis to clinical nutrition administration below or above the first quartile, respectively. [2] (First row) Proportions calculated over the total number of metastatic patients who received clinical nutrition. (Following rows) Proportions calculated over the total number of metastatic patients with the corresponding cancer type who received clinical nutrition. [3] (First row) Proportions calculated over the total number of metastatic patients who received early clinical nutrition. (Following rows) Proportions calculated over the total number of metastatic patients with the corresponding cancer type who received early clinical nutrition. [4]mCCI. Modified Charlson Comorbidity Index not accounting for cancer. [5](First row) Proportions calculated over the total number of metastatic patients who received late clinical nutrition. (Following rows) Proportions calculated over the total number of metastatic patients with the corresponding cancer type who received late clinical nutrition.

Table S2. Results from Cox multivariate regression models comparing death risk in metastatic patients receiving early versus late clinical nutrition.

	**Head and Neck (*N* = 152)**	**Gastrointestinal (*N* = 1478)**	**Respiratory (*N* = 389)**	**Genitourinary (*N* = 312)**
	HR [95% CI]	HR [95% CI]	HR [95% CI]	HR [95% CI]
Clinical nutrition (early VS late)	1.3 [0.6 – 2.7]	0.5 [0.4 – 0.6]*	0.5 [0.3 – 0.9]*	0.6 [0.3 – 0.9]*
Gender (male VS female)	1.3 [0.6 – 2.5]	0.9 [0.8 – 1.1]	1.2 [0.9 – 1.6]	0.9 [0.7 – 1.3]
Age	1.0 [1.0 – 1.1]	1.0 [1.0 – 1.0]*	1.0 [1.0 – 1.0]	1.0 [1.0 – 1.0]
mCCI	0.8 [0.6 – 1.2]	0.9 [0.9 – 1.0]	0.9 [0.8 – 1.0]	0.9 [0.7 – 1.1]
Chemotherapy (yes VS no)	0.59 [0.3 – 1.1]	0.9 [0.7 – 1.0]	1.1 [0.8 – 1.5]	1.0 [0.7 – 1.3]

* p-Value < 0.05.

Table S3. Differential diagnoses of malnutrition in the cohort of metastatic patients with CN (a) and without CN (b).

**(a) Malnourished metastatic patients with CN**								
**Malnutrition condition (ICD-9-CM code)**	**Head and Neck (*N* = 51)**		**Gastrointestinal (*N* = 347)**		**Respiratory (*N* = 74)**		**Genitourinary (*N* = 51)**	
	**N**	**%**	**N**	**%**	**N**	**%**	**N**	**%**
Kwashiorkor (260)	0	0.0	NI	-	0	0.0	0	0.0
Nutritional marasmus (261)	4	7.8	33	9.5	7	9.5	5	9.8
Other severe protein–calorie malnutrition (262)	20	39.2	94	27.1	26	35.1	16	31.4
Other and unspecified protein–calorie malnutrition (263)	33	64.7	209	60.2	42	56.8	32	62.7
Vitamin A deficiency 264	0	0.0	0	0.0	0	0.0	0	0.0
Thiamine and niacin deficiency states (265)	0	0.0	0	0.0	0	0.0	0	0.0
Deficiency of B-complex components (266)	0	0.0	NI	-	0	0.0	NI	-
Ascorbic acid deficiency (267)	0	0.0	0	0.0	0	0.0	0	0.0
Vitamin D deficiency (268)	0	0.0	4	1.2	NI	-	NI	-
Other nutritional deficiencies (269)	0	0.0	41	11.8	5	6.8	NI	-
**(b) Malnourished metastatic patients without CN**								
**Malnutrition condition (ICD-9-CM code)**	**Head and Neck (*N* = 85)**		**Gastrointestinal (*N* = 387)**		**Respiratory (*N* = 191)**		**Genitourinary (*N* = 138)**	
	**N**	**%**	**N**	**%**	**N**	**%**	**N**	**%**
Kwashiorkor (260)	0	0.0	NI	-	0	0.0	NI	-
Nutritional marasmus (261)	5	5.9	33	8.5	14	7.3	14	10.1
Other severe protein–calorie malnutrition (262)	30	35.3	122	31.5	68	35.6	38	27.5
Other and unspecified protein–calorie malnutrition (263)	47	55.3	148	38.2	72	37.7	60	43.5
Vitamin A deficiency 264	0	0.0	NI	-	0	0.0	0	0.0
Thiamine and niacin deficiency states (265)	0	0.0	NI	-	0	0.0	0	0.0
Deficiency of B-complex components (266)	9	10.6	31	8.0	14	7.3	9	6.5
Ascorbic acid deficiency (267)	0	0.0	0	0.0	0	0.0	0	0.0
Vitamin D deficiency (268)	NI	-	40	10.3	13	6.8	12	8.7
Other nutritional deficiencies (269)	NI	-	30	7.8	15	7.9	11	8.0

Following the "Opinion 05/2014 on Anonymisation Techniques" drafted by the "European Commission Article 29 Working Party", the analyses involving less than 3 patients were not reported, as potentially reconductable to single individuals. Therefore, results referred to ≤ 3 patients were reported as NI (not issuable). The diagnoses are not mutually exclusive

Table S4. Results from Cox multivariate regression models comparing death risk in malnourished metastatic patients with clinical nutrition versus without clinical nutrition.

	**Head and Neck (*N* = 136)**	**Gastrointestinal (*N* = 734)**	**Respiratory (*N* = 265)**	**Genitourinary (*N* = 189)**
	HR [95% CI]	HR [95% CI]	HR [95% CI]	HR [95% CI]
Clinical nutrition (yes VS no)	1.0 [0.7 – 1.6]	0.8 [0.6 – 0.9]*	0.7 [0.5 – 1.0]	0.6 [0.4 – 1.0]*
Gender (male VS female)	1.9 [1.1 – 3.2]*	1.2 [1.0 – 1.4]*	1.1 [0.8 – 1.5]	1.2 [0.8 – 1.6]
Age	1.0 [1.0 – 1.0]	1.0 [1.0 – 1.0]*	1.0 [1.0 – 1.0]*	1.0 [1.0 – 1.1]*
mCCI	0.9 [0.7 – 1.2]	1.0 [0.9 – 1.1]	0.9 [0.8 – 1.0]	1.0 [0.9 – 1.2]
Chemotherapy (yes VS no)	0.6 [0.4 – 0.9]*	0.7 [0.6 – 0.8]*	0.7 [0.5 – 1.0]*	0.6 [0.4 – 0.8]*

* p-Value < 0.05.

Table S5. Characteristics of non-metastatic patients who received clinical nutrition stratified by cancer type.

	**Non-metastatic patients with clinical nutrition (N= 4,379)**				
	**Age Mean (SD)**	**Men *N* (%)**	**mCCI[1] Mean (SD)**	**Malnutrition *N* (%)**	**Chemotherapy *N* (%)**
All Clusters (*N* = 4,379)	70.3 (16.1)	2,678 (61.2)	1.3 (1.3)	613 (14.0)	390 (8.9%)
Head and Neck (*N* = 202)	69.0 (12.3)	130 (64.4)	1.2 (1.1)	47 (23.3)	8 (4.2)
Gastrointestinal (*N* = 2,114)	74.1 (11.6)	1,212 (57.3)	1.4 (1.4)	381 (18.0)	21 (1.0)
Respiratory (*N* = 405)	72.2 (12.5)	323 (79.8)	1.6 (1.1)	46 (11.4)	5 (1.3)
Genitourinary (*N* = 604)	76.3 (12.9)	431 (71.4)	1.6 (1.3)	58 (9.6)	11 (1.9)
Hematology (*N* = 1054)	58.9 (21.1)	582 (55.2)	0.9 (1.2)	83 (7.9)	314 (29.8)

[1]mCCI. Modified Charlson Comorbidity Index not accounting for cancer.

Table S6. Results from Cox multivariate regression models on non-metastatic patients comparing death risk in non-metastatic patients receiving early versus late clinical nutrition.

	**Head and Neck (*N* = 135)**	**Gastrointestinal (*N* = 1490)**	**Respiratory (*N* = 264)**	**Genitourinary (*N* = 453)**	**Hematology (*N* = 785)**
	HR [95% CI]	HR [95% CI]	HR [95% CI]	HR [95% CI]	HR [95% CI]
Clinical nutrition (early VS late)	0.6 [0.3– 1.0]	0.8 [0.6 – 1.0]*	0.7 [0.5 – 1.1]	1.0 [0.7 – 1.5]	0.8 [0.6 – 1.1]
Gender (male VS female)	2.7 [1.4– .4]*	1.2 [1.0 – 1.5]	0.9 [0.5 – 1.6]	0.8 [0.6 – 1.2]	1.0 [0.7 – 1.3]
Age	1.0 [1.0 – 1.1]*	1.0 [1.0 – 1.0]*	1.0 [1.0 – 1-0]*	1.0 [1.0 – 1-1]*	1.0 [1.0 – 1.0]*
mCCI	1.3 [1.0 – 1.6]	1.1 [1.0 – 1.2]*	1.1 [0.9 – 1.3]	1.1 [0.9 – 1.2]	1.0 [0.9 – 1.1]
Chemotherapy (yes VS no)	0.8 [0.3 – 1.8]	0.8 [0.6 – 1.1]	1.5 [0.8 – 2.8]	0.2 [0.0 – 1.7]	1.2 [0.9 – 1.7]

* p-Value < 0.05.
